# The Andersen Aerobic Fitness Test: Reliability and Validity in 10-Year-Old Children

**DOI:** 10.1371/journal.pone.0110492

**Published:** 2014-10-17

**Authors:** Eivind Aadland, Torkil Terum, Asgeir Mamen, Lars Bo Andersen, Geir Kåre Resaland

**Affiliations:** 1 Sogn og Fjordane University College, Faculty of Health Studies, Førde, Norway; 2 Sogn og Fjordane University College, Faculty of Teacher Education and Sports, Sogndal, Norway; 3 University College of Health Sciences – Campus Kristiania, Oslo, Norway; 4 Department of Sports Medicine, Norwegian School of Sport Sciences, Oslo, Norway; 5 Department of Sport Sciences and Clinical Biomechanics, University of Southern Denmark, Odense, Denmark; 6 Sogn og Fjordane Center for Health Research, Førde Central Hospital, Førde, Norway; Louisiana State University, United States of America

## Abstract

**Background:**

High aerobic fitness is consistently associated with a favorable metabolic risk profile in children. Direct measurement of peak oxygen consumption (VO_2peak_) is often not feasible, thus indirect tests such as the Andersen test are required in many settings. The present study seeks to determine the reliability and validity of the Andersen test in 10-year-old children.

**Methods:**

A total of 118 10-year-old children (67 boys and 51 girls) were recruited from one school and performed four VO_2peak_ tests over three weeks: three Andersen tests (indirect) and one continuous progressive treadmill test (direct). Of these, 104 children provided valid data on all Andersen tests and 103 children also provided valid data on the direct treadmill test. Reliability and validity were assessed using Bland Altman plots and linear regression analysis.

**Results:**

Bias (mean change) and random error (limits of agreement) were 26.7±125.2 m for test 2 vs. test 1 (p<.001 for mean difference) and 3.9±88.8 m for test 3 vs. test 2 (p = .514 for mean difference). The equation to estimate VO_2peak_ suggested by Andersen et al. (2008) showed a poor fit in the present sample; thus, we suggest a new equation: VO_2peak_ = 23.262+0.050*Andersen distance –3.858*gender –0.376*body weight (R^2^ = 0.61, standard error of the estimate = 5.69, p<.001, boys = 0, girls = 1).

**Conclusions:**

The Andersen test provided reliable and valid data on a group level. However, a substantial degree of individual variability was found for estimates of VO_2peak_. Researchers should be aware of the amount of noise in indirect tests that estimate aerobic fitness.

## Introduction

High aerobic fitness is consistently associated with a favorable metabolic risk profile in children [1], [2]. In adults, the relationship between aerobic fitness and health becomes evident through increased incidence of cardiovascular disease and mortality in those having a poor fitness level compared to their more fit peers [3], [4]. In order to inform the society regarding targets for public health management in childhood and to determine effective interventions in this population, being able to measure aerobic fitness in a valid and reliable way in relatively large groups of children (e.g. a school setting) is a prerequisite.

The most-used criterion measure for maximal aerobic fitness is maximal (peak) oxygen consumption measured to voluntary exhaustion during an incremental treadmill or bicycle protocol. However, such testing is time-consuming and requires expensive equipment and highly trained test personnel, and would therefore not be feasible for testing large samples of children (e.g. school classes). Therefore, both maximal and submaximal performance measures have been developed to estimate VO_2max_ for use with different groups in various settings [5]. A highly used test in children is the 20 m multistage shuttle run test (MSRT) [6], [7]. However, recent external validation studies have shown that current equations to estimate VO_2peak_ in children 8–13 years old may be questionable [8], [9] due to biased estimates and large individual errors. In addition, the MSRT test protocol has some drawbacks, especially when applied for children. Therefore, an alternative test was proposed by Andersen et al. [10] and is now included as a measure of aerobic fitness in several large studies [11]–[13]. The Andersen test is an intermittent running test (15 seconds working, 15 seconds resting) using a 20 m lane, where children aim to cover as long a distance as possible during 10 minutes. Compared to the MSRT, the Andersen test may have several advantages: 1) it relates closer to children’s usual running pattern (i.e. intermittent vs. continuous activity), 2) it does not stigmatize children having poor fitness and therefore does not exclude them early from the test (i.e. all children finish the test at their own maximal pace independent of fitness level), and 3) it does not require any equipment besides a stopwatch, measuring tape, and a whistle. However, besides the original study by Andersen et al. [10] and a small study by Ahler et al. [14] (in children 6–9 years old), the measurement properties of the Andersen test have not been thoroughly examined.

The present study seeks to determine the reliability and criterion validity of the Andersen test in a relatively large sample (n>100) of 10-year-old children. Reliability was assessed using three admissions of the Andersen test over three weeks. Validity was assessed using VO_2peak_ determined from a maximally graded treadmill protocol as the criterion measure.

## Materials and Methods

### Ethics statement

Children and their parents were given thorough oral and written information regarding the study protocol. Each child orally agreed to participate in the study, and written informed consent was obtained from each child’s parent(s)/guardian(s) prior to the child’s inclusion in the study. The study met the standards of the Declaration of Helsinki and was approved by the Regional Committee for Medical Research Ethics (REC West) in Norway.

### Subjects

All 121 children in fifth grade (10-year olds) at one school in the western part of Norway over two consecutive school years were invited to participate in the study. A total of 118 children (67 boys and 51 girls; 58 during 2012–2013 and 60 during 2013–2014) were included in the study. Three children were excluded from the study (one child performed the testing, but were excluded for being severely short of growth; two children did not perform the testing for medical reasons (one for having heart problems and one for having a skeletal disease).

### Study protocol

Children performed three Andersen tests (weeks 1, 3, and 4), and performed one incremental treadmill test to exhaustion (week 2) to measure their peak oxygen consumption (VO_2peak_) within three weeks. Children were instructed not to eat during the last two hours prior to testing and to engage in normal physical activity the day before the test and the day of testing.

The Andersen test was performed according to standard procedures [10]. Two parallel lines 20 m apart were marked in a gym hall with a wooden floor. The children were informed about the procedures and performed a collective five-minute warm-up before the test. The test has a total duration of 10 minutes, where children run from one end line to another in a to-and-fro movement intermittently, with 15-second work periods and 15-second breaks signaled by the test leader’s blowing a whistle. When the children finished one 15-second period of work, they were instructed to stop as fast as possible and to take one to three steps back, depending on how fast they were able to stop. Each time the children turned around at an end line, they had to touch with one finger the floor behind the end line. The goal was to cover the longest possible distance during the 10-minute run. Verbal encouragement was highly standardized across all tests. It was kept to a minimum during the first half of the test and increased gradually and consistently toward the test’s final part. The distance covered (number of laps performed) was recorded by adult test assistants who counted for one or two children each. Each of the two classes was split into three subgroups for testing (according to how their classes were usually divided in school), leaving approximately 20 children per test. The gym hall was 18.1 m wide, giving each child a lane of about 1 m.

Peak oxygen consumption was measured to exhaustion using an incremental treadmill test. The treadmill’s inclination (Woodway PPS 55, Woodway GmbH, Weil am Rhine, Germany) was constant at 5.3% during the whole test. Children started to walk at 5 km/h for 5 minutes. Thereafter the speed increased by 1 km/h each minute until the children were exhausted. Oxygen consumption was measured using the Moxus Modular Metabolic System (AEI Technologies Inc., Pittsburgh, USA). A two-point gas calibration according to known concentrations and calibration according to atmospheric pressure were performed each test day. Volume calibration of the breathing valve (Hans Rudolph model 2700, Hans Rudolph Inc., Shawnee, Kansas, USA) was performed between each test using a 3-l syringe (Series 5530, Hans Rudolph, Kansas, USA). The oxygen analyzer has shown to be reliable and valid compared to the Douglas-bag technique [15]. To prevent injuries in case of falls during the test, children performed the test with a safety rope connected to a chest-belt system from Cosmos (h/p/cosmos sports & medical GmbH, Nussdorf-Traunstein, Germany). Throughout the test, a test assistant was in charge of the subject’s safety by tightly holding the safety rope. If the subject stumbled, the test assistant could pull the rope, thereby raising the subject and preventing a fall. The child and parent(s)/guardian(s) were informed of test procedures before testing, and the child’s parent(s)/guardian(s) were allowed and encouraged to observe the testing.

After each test, test leader and associates discussed several subjective criteria to verify a near maximal performance: hyperpnoea, unsteady running pattern, and verbal and body language clearly indicating that the child wanted to stop testing despite repeated strong verbal encouragement. Additionally, the respiratory exchange ratio (RER) and maximal heart rate (HR_peak_) (Polar S610i HR monitor, Polar Electro OY, Kempele, Finland) were noted. The reliability of VO_2peak_ tested directly in children is shown to be approximately 4%, which compares favorably with the reliability of testing of adults’ VO_2max_ [16].

The VO_2peak_ is presented as absolute (l/min) and relative values (ml/kg/min), each of which is defined as the highest value of two successive 30-second measurements. Height and body weight were measured without shoes and socks before the children started the VO_2peak_ test. Height was measured to the nearest 0.1 cm using a wall-mounted stadiometer. Body weight was measured to the nearest 0.1 kg (subtracting 0.2 kg for light clothes) using an electronic scale (Seca 770, SECA GmbH, Hamburg, Germany). Body weight was used as a continuous variable in the statistical analyses. For the purpose of reporting of descriptive statistics, children were also categorized as normal weight, overweight, or obese according to the criteria set by Cole et al. [17].

### Statistical analyses

The anthropometric subject characteristics and data on VO_2peak_ and the Andersen test are presented as the mean values and standard deviation (SD).

Reliability of the Andersen test was assessed by determining a) bias (learning effect) and b) random errors over the three tests. Bias was determined using a linear mixed model that included a random intercept for subjects. Test number was included as a fixed-effect factor variable. The effect of gender was tested by adding the main effect and an interaction term (gender*test number) to the model. Effects are reported as effects estimates and 95% confidence intervals (CI). Random error was determined using Pearson correlation (r), intraclass correlation coefficient (ICC_3,1_), and Bland Altman plots. The Bland Altman plots show the difference between two subsequent tests as a function of the mean of the two tests [18]. Because the data were deemed to be homoscedastic, the limits of agreement (LoA) were calculated according to Hopkins [19] (LoA = SD of the differences*1.96).

Validity of the Andersen test was assessed using Pearson’s r, linear regression, and Bland Altman plots in three steps: 1) We applied the suggested equation (VO_2max_ = 18.38+0.033*Andersen distance –5.92*gender [boys = 0; girls = 1]) from Andersen et al. [10] to predict VO_2peak_ in our sample. 2) To develop a new equation to predict VO_2peak_ from the Andersen test, we initially split our sample in two to perform a validation of our equation in an independent dataset. The children included during 2012–2013 served as the training dataset (n = 52) from which the equation was developed, whereas the group included during 2013–2014 served as the testing dataset (n = 51) Three variables were included in the model (VO_2peak_ = a+b*Andersen distance+c*gender+d*body weight, [boys = 0; girls = 1]). The predicted and measured VO_2peak_ were then compared using linear regression and a Bland Altman plot. Means were compared using a one-sample t-test. 3) Finally, we calculated a new equation based on the whole sample (n = 103) using the following model (VO_2peak_ = a+b*Andersen distance+c*gender+d*body weight, [boys = 0; girls = 1]). The final model is reported as regression coefficients with 95% bootstrapped CIs. The better of the Andersen tests 1 and 2 was used in all analyses of validity.

All analyses were performed using IBM SPSS v. 20 (IBM Corporation, Software Group, Somers, New York, USA). A p-value<.05 indicated statistically significant findings.

## Results

### Children’s characteristics

Except for a significantly higher VO_2peak_ in boys than in girls (p<.001), there were no significant differences between genders or the two subsamples included (p>.095) (table 1). Of the total sample included (n = 118), 113, 112, and 112 children provided valid data for the Andersen test numbers 1, 2, and 3, respectively. A total of 104 children provided valid data on all Andersen tests and were included in analyses of reliability. Reasons for not undertaking the test were sickness or being out of school (n = 3, 4, and 4 at tests 1, 2, and 3, respectively), whereas reasons for not providing a valid test were falls and complaints about being uncomfortable (nausea or musculoskeletal pain) (3, 2, and 2 at tests 1, 2, and 3, respectively). Of the children reporting musculoskeletal pain, the pain for two individuals was directly related to the test performed (one fell and hit his knee in the floor; one suffered an acute ankle sprain). A total of 113 children provided valid data on directly measured VO_2peak_ on the graded treadmill protocol (two children did not perform the test, one child was excluded for not performing a maximal test, and two children were excluded due to technical errors). Of these, 103 children provided valid data on Andersen test numbers 1 and 2 (the better of performance 1 or 2 was used for the purpose of analyzing validity); thus, 103 children were included in the analyses of validity.

**Table 1:** Children’s characteristics (mean (SD)).

	Overall	Boys	Girls	Year 1	Year 2
Number (%)	118	67 (57)	51 (43)	58 (49)	60 (51)
Age (years)	10.3 (0.3)	10.3 (0.3)	10.3 (0.3)	10.3 (0.3)	10.2 (0.3)
Height (cm)	143.4 (5.8)	143.9 (6.2)	142.7 (5.1)	143.5 (5.5)	143.3 (6.0)
Body weight (kg)	38.0 (8.4)	38.3 (9.5)	37.6 (6.8)	38.2 (9.0)	37.7 (8.0)
Body mass index (kg/m^2^)	18.4 (3.2)	18.3 (3.6)	18.4 (2.7)	18.4 (3.3)	18.3 (3.2)
% overweight/obese	20.3	20.9	19.6	22.4	18.3
Waist circumference (cm)	64.3 (8.6)	64.9 (9.5)	63.6 (7.3)	63.9 (8.6)	64.7 (8.8)
VO_2peak_ (l/min)*	2.00 (0.34)	2.10 (0.35)	1.88 (0.29)	2.06 (0.33)	1.96 (0.35)
VO_2peak_ (ml/kg/min)*	54.0 (9.0)	56.6 (9.1)	50.5 (7.5)	55.4 (9.5)	52.6 (8.2)

*overall n = 113; n = 66 for boys and 47 for girls; n = 57 for first year and 56 for second year.

Maximal heart rate and respiratory exchange ratio on the VO_2peak_ test were (mean (SD)) 201 (8.9) beats/minute and 1.07 (0.07), respectively.

### Reliability

Running distance on the Andersen test across the three tests is shown in table 2. Running distance increased significantly from test 1 to test 2 (mean (95% CI) 26.7 (14.8 to 38.6) m or 3.0 (1.6 to 4.3)%, p<.001), whereas no difference was found between test 2 to test 3 (3.9 (−8 to 15.8) m, p = .514). Boys ran 59.2 (20.5 to 97.9) m farther than girls did (p = .003). However, the initial increase and thereafter a plateau in performance were similar between genders (p for gender*test number = .189). Overall, 19 (18%), 38 (37%), and 47 (45%) children achieved their longest running distance (personal best) on tests 1, 2, and 3, respectively.

**Table 2:** Running distance (m) on the three Andersen tests (mean (SD)).

Test number	Overall(n = 104)	Boys(n = 61)	Girls(n = 43)	Year 1(n = 51)	Year 2(n = 53)
1	897 (111)	918 (126)	867 (79)	923 (102)	871 (115)
2	923 (99)	946 (108)	891 (74)	957 (104)	891 (84)
3	927 (112)	957 (117)	885 (92)	968 (106)	888 (105)
Better of 1 and 2	935 (93)	958 (102)	902 (69)	965 (95)	906 (83)
Overall best	947 (98)	974 (104)	909 (73)	978 (100)	919 (86)

**Figure 1:**
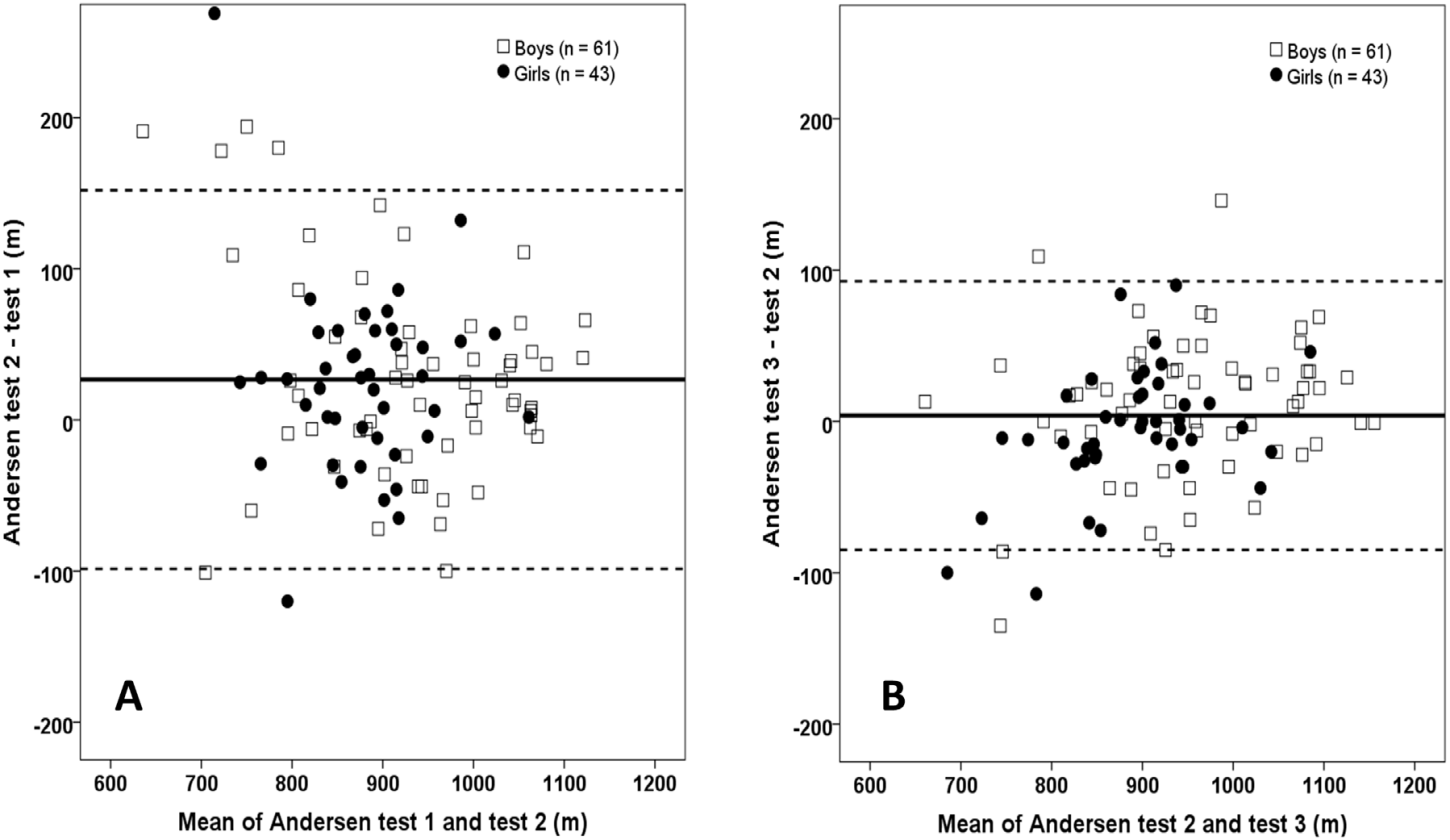
Bland Altman plots showing differences between test 1 and test 2 (A) and between test 2 and test 3 (B) as a function of the mean of the two corresponding tests. The solid line is the mean difference; dotted line is limits of agreement (bias±1.96*SD of the difference).

The relationship between all Andersen tests was high and similar between genders (test 1 vs. 2: r = 0.82; test 1 vs. 3: r = 0.79; test 2 vs. 3: r = 0.92, best of tests 1 and 2 vs. overall best: r = 0.97, all p<.001) having an overall ICC_3,1_ = 0.84 (CI 0.78 to 0.88). Still, the Bland Altman plots (figure 1) reveal some individual variability in distance covered between test 1 and test 2 (bias ± LoA = 26.7±125.2 m (LoA = 13.9% of test 1 mean)), but smaller for test 2 vs. test 3 (3.9±88.8 m (LoA = 9.6% of test 2 mean)).

### Validity and prediction equation

The bivariate relationships between the Andersen tests and VO_2peak_ were r = 0.63, r = 0.70, r = 0.68, r = 0.72, and r = 0.73 for Andersen tests 1, 2, and 3, the best of tests 1 and 2, and the overall best test vs. VO_2peak_, respectively (n = 100, i.e. those having valid data on all of these measurements).

**Figure 2:**
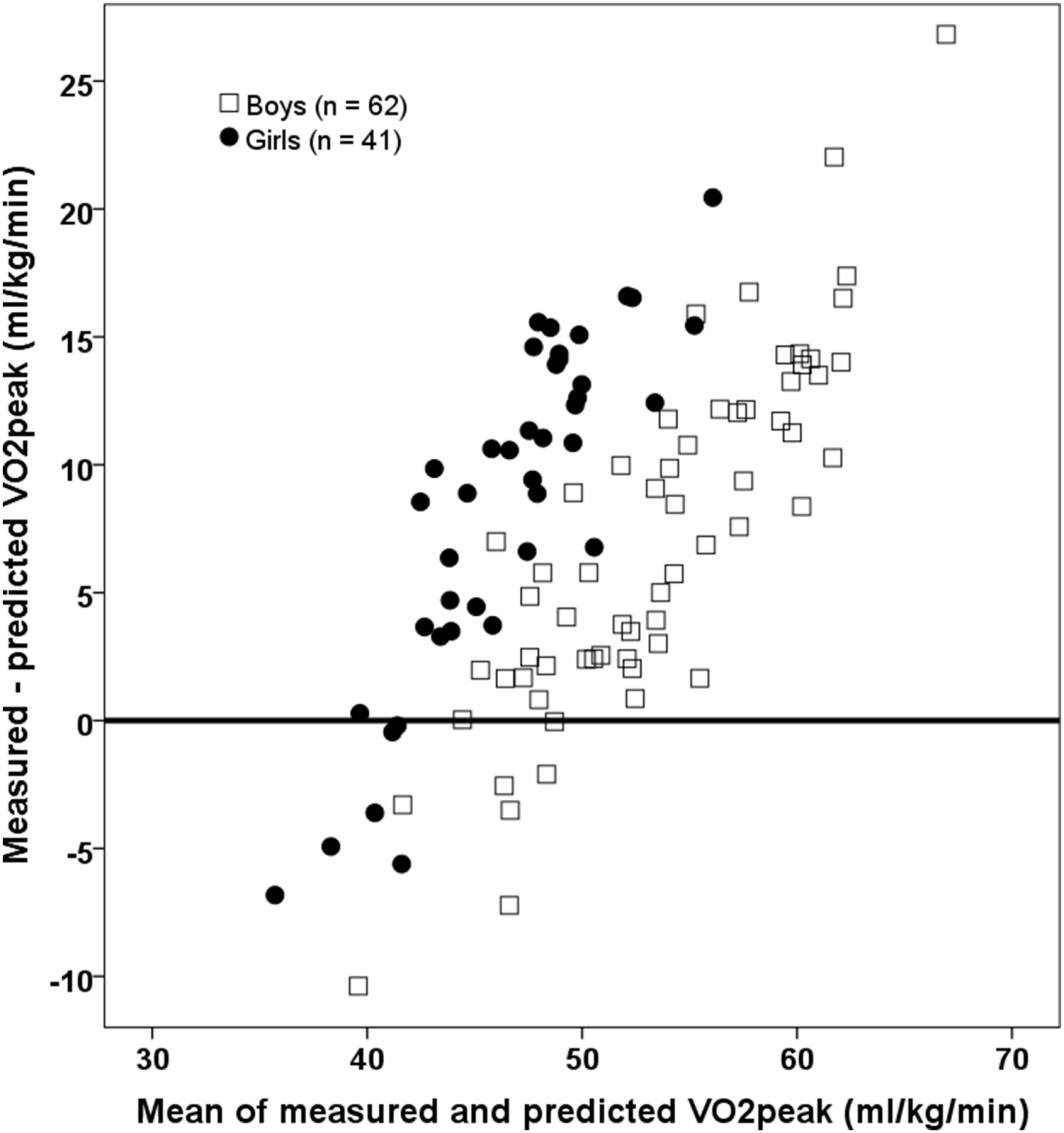
Bland Altman plots showing differences between measured VO_2peak_ and predicted VO_2peak_ from Andersen et al. (VO_2peak_ = 18.38+0.033*Andersen test –5.92*gender) as a function of the mean of the two values.

The equation to predict VO_2peak_ suggested by Andersen et al. [10] was clearly inadequate to predict VO_2peak_ in the present sample (slope for mean vs. differences of measured vs. predicted VO_2peak_: p<.001). Figure 2 shows that the Andersen et al. equation severely and systematically underestimates VO_2peak_ (mean ± SD 46.9 (4.8) vs. 54.5 (9.0) ml/kg/min, p<.001), especially for children having above median VO_2peak_ values. Thus, a new equation would be required to better fit our data.

**Figure 3:**
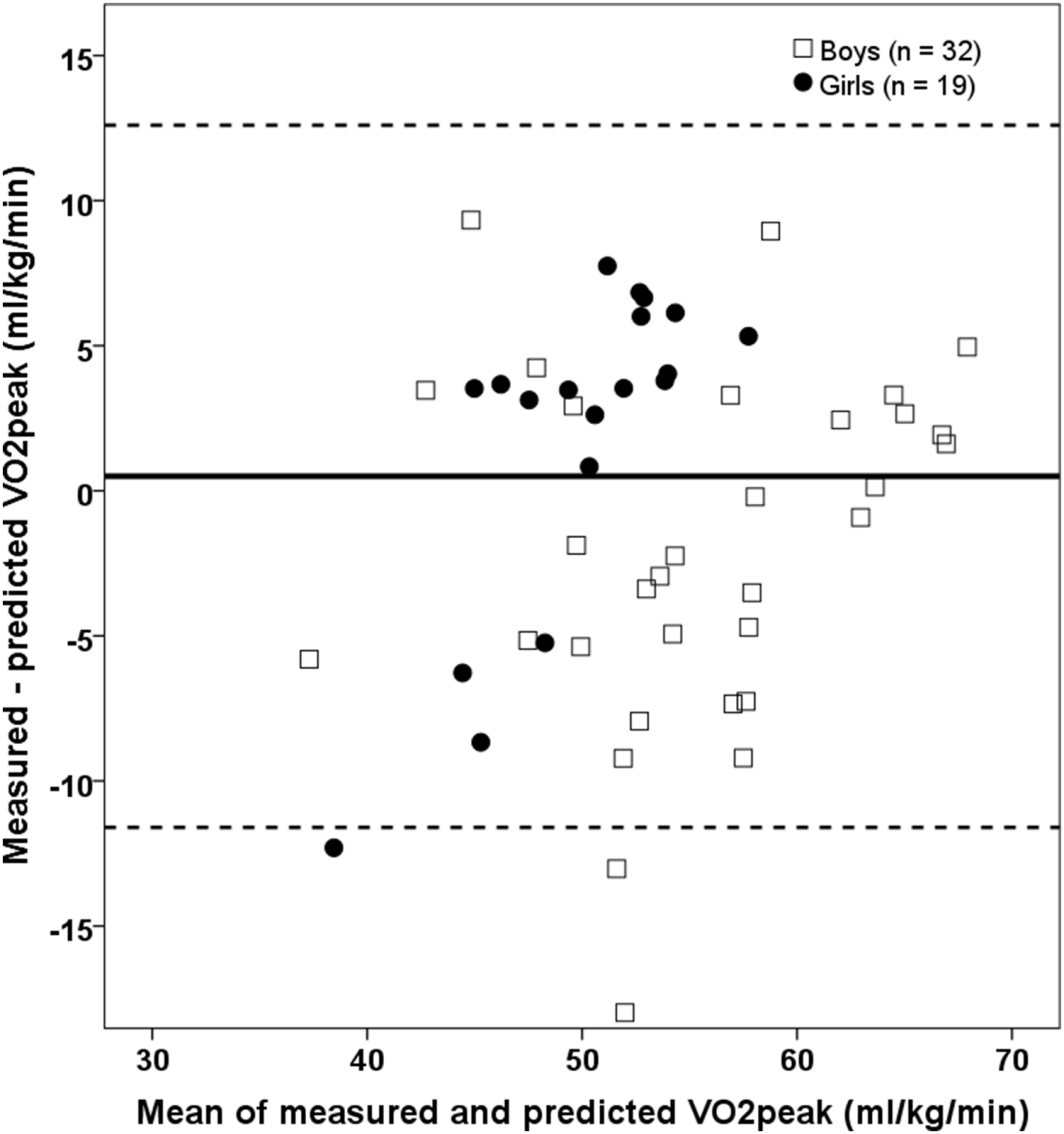
Bland Altman plots showing differences between measured VO_2peak_ and predicted VO_2peak_ as a function of the mean of the two values in class 2 (testing dataset) based on the regression equation derived from class 1 (training dataset) (VO_2peak_ = 22.887+0.052*Andersen test –5.632*gender –0.386*body weight). The solid line is the mean difference; dotted line is limits of agreement (bias±1.96*SD of the difference).

To develop a new equation to predict VO_2peak_ from the Andersen test, we initially split our sample in two groups (a training dataset including 52 children and a testing dataset including 51 children) to perform a validation of our equation in an independent dataset. The equation developed was as follows (regression coefficients and 95% CI): VO_2peak_ = 22.887 (−0.591 to 46.365)+0.052 (0.033 to 0.071)*Andersen distance –5.632 (−8.774 to −2.491)*gender –0.386 (−0.598 to −0.174)*body weight (R^2^ = 0.71, standard error of the estimate (SEE) = 5.37, p<.001, boys = 0, girls = 1). Predicted vs. measured VO_2peak_ yielded R^2^ = 0.46 and SEE = 6.06. Mean values were close to identical between predicted and measured VO_2peak_ (53.6 (7.0) vs. 53.1 (8.2) ml/kg/min, p = .514). Limits of agreement between the predicted and measured values were±12.1 ml/kg/min (±22% of mean VO_2peak_), indicating a relatively large degree of uncertainty on individual-level predictions (figure 3).

As the derived equation performed sufficiently in the independent testing set, we established a new equation based on the whole sample (regression coefficients and 95% bootstrapped CI): VO_2peak_ = 23.262 (4.934 to 39.694)+0.050 (0.038 to 0.063)*Andersen distance –3.858 (−6.106 to −1.539)*gender –0.376 (−0.509 to −0.248)*body weight (R^2^ = 0.61, SEE = 5.69, p<.001, boys = 0, girls = 1, n = 103).

## Discussion

The present study’s main finding was that the Andersen test is a reliable and valid tool for determination of aerobic fitness on a group level. However, a substantial degree of individual variability should be expected for estimates of VO_2peak_ based on the Andersen test. Moreover, at least two Andersen tests should be performed to obtain valid results.

We detected an increased running distance of 3% from test 1 to test 2. This increase is in contrast to previous studies showing no significant bias over subsequent tests examining the Andersen test [10], [14] and the MSRT [20]–[23] in children and youth. Our estimate shows that one should expect the distance ran to increase 15–39 meters (1.6–4.3%) from the first to the second test admission on a group level. This improvement equals an increased VO_2peak_ of 1.3 (95% CI 0.7 to 1.9) ml/kg/min, when the suggested equation from the present study is used to estimate it. There was no further increase in distance ran to the third test admission. These results indicate that one familiarization trial prior to the test admission, or using the better of two tests, would be recommended to avoid any learning effect that might invalidate the test results. This finding is in line with findings from a study examining three admissions of the MSRT in adults [24]. Still, the bias may be interpreted as relatively minor, and might also be adjusted based on the current findings.

However, if the test is used to evaluate the aerobic fitness of individual children (e.g. evaluation of children in a practical school setting or if regression analysis is run on the subject level), some variation from test to test must be expected. Despite the high correlation found between test 2 and test 3 (r = 0.92), individual differences would be expected to be from −85 to 93 m (±10% of the mean performance) between these tests. However, using the better result of two tests will provide researchers with a precise estimate of the Andersen test’s performance (r = 0.97 with the overall best test).

As reliability is a premise for validity, variation in test performance over time will weaken the “real” relationship with VO_2peak_, as noise in the predictor (x-variable) induces regression dilution bias [25]. The amount of random error decreased over time (test 2 vs. test 1 compared to test 3 vs. test 2) in the present study. Thus, the use of a second or third test, or the best of two or more tests, will increase both reliability and validity of the test. Although a marginally increased fit was found in the present study using the best Andersen test compared to one single test (results not shown), others have shown clear improvement in predictive ability for VO_2peak_ using the overall best test [20]. Nevertheless, although each researcher must ultimately decide whether a measurement tool is reliable, given the purpose of the study [19], [26], we believe the Andersen test, given the use of a familiarization trial or the best of two or more scores, provides reliable data regarding a child’s level of aerobic fitness.

Estimation of VO_2peak_ based on the equation suggested by Andersen et al. [10] yielded a poor fit in our sample due to a lower slope than that of the present study. This picture is very similar to that of studies that externally validated various equations for the MSRT [8], [9]. Although such systematic differences between measured and predicted VO_2peak_ possibly could be explained by many factors, there are few clear answers. An obvious reason for a biased equation (slope) is inherent (random) variation in the examined relationship from study to study, especially when the dataset upon which the equation is based (or the equation is tested) is small. In the present study, regression coefficients from 0.022 to 0.054 (CIs 0.004 to 0.080) (results not shown) were found across the three Andersen tests in the two classes, despite a more or less identical setting across all tests (children from the same population, same procedures, same testers). The bias could also be caused by a confounding variable that may change across samples, time, places, and testers (i.e. the model is underfitted and fails to account for an important variable). Equations for the MSRT vary in their included variables – some include age, gender, and a measure of body fat [8], [9]. Andersen et al. [10] did not include body fat or weight in their original equation to estimate VO_2peak_, however, body weight was a highly significant predictor in the present study. Moreover, body weight was significantly (p<.001) related to the difference between measured VO_2peak_ and VO_2peak_ predicted from Andersen et al. (result not shown), which may indicate that the original model was underfitted.

To create a new equation, we performed an external validation within our sample (using a training dataset and an independent test dataset) prior to establishing the final equation [27]. The procedure showed no bias, and no significantly different slope between the predicted and measured VO_2peak_ in the test dataset. This lack of both slope difference and bias indicated that the equation was sufficiently stable for estimating VO_2peak_ in an independent sample. However, consistent with previous studies [8], [9], we found quite large limits of agreement for estimates on an individual level. Our results showed that one must expect individual deviations in VO_2peak_ of ±12 ml/kg/min (±22% of mean VO_2peak_) based on the Andersen test, gender, and body weight. This level of deviation limits the usefulness of the Andersen test to estimate VO_2peak_ on an individual basis. Batista et al. [9] and Melo et al. [8], who found LoAs of similar size for estimating VO_2peak_ from the MSRT in external validation studies, both concluded that the test is unsuitable to estimate VO_2peak_ on an individual level. We do not believe such tests are unsuitable for individual predictions; however, we agree that researchers should be aware of the amount of noise in these indirect tests, because it may greatly dilute any relationship between aerobic fitness and health [25]. Thus, future studies should directly compare the use of direct and indirect measures of aerobic fitness regarding their ability to predict health outcomes.

### Strengths and limitations

The present study has two main strengths. First, we included a relatively large sample of children, which made it possible to perform an external validation of our equation for VO_2peak_ and to arrive at relatively stable estimates for VO_2peak_, compared to many other studies that are based on small sample sizes. Second, we included three admissions of the Andersen test, an approach which allowed us to evaluate the performance difference between test 2 and test 3. None of the previous studies of the Andersen test included more than two admissions [10], [14].

Limitations of the study are related to the sample included. One could argue that our test dataset was not fully independent, as the children composing both the training and test datasets came from the same school and performed the tests in the same gym hall, led by the same testers [27]. Thus, the equation could be expected to perform worse in other contexts, and further external validation studies are desired. Moreover, our sample was restricted to 10-year-old children. Although Andersen et al. [10] did not find any age-specific relationship with directly measured VO_2peak_ in their original study, running economy improves with age [28]; thus, the equation suggested to estimate VO_2peak_ in the present study may not be valid in other age groups.

### Conclusions

We conclude that the Andersen test provided reliable and valid data on a group level for 10-year-old children. However, a substantial degree of individual variability was found for estimates of VO_2peak_ based on the Andersen test. Researchers should be aware of the amount of noise in the Andersen test and in other indirect tests to estimate aerobic fitness, because “real” relationships between aerobic fitness and health are diluted and increase the probability of performing type II errors. In any case, we recommend that a familiarization trial or several Andersen tests be performed to increase the precision of the measurement.

## Supporting Information

Data File S1 **Supplementary data file including all material underlying the present study.**
(SAV)
